# Agreement Between Carrea’s Odontological Method and De Mendonça’s Osteometric Method for Stature Estimation: A Study on Skeletal Remains from an Andalusi Necropolis (Zaragoza,Spain)

**DOI:** 10.3390/life16091514

**Published:** 2026-09-11

**Authors:** Ana Isabel Cisneros-Gimeno, Annika Fieguth-Batista, Miriam Gracia-Martínez, Jésus Obón-Nogués, Jaime Whyte-Orozco, Alberto García-Barrios

**Affiliations:** 1 Department of Human Anatomy and Histology, School of Medicine, University of Zaragoza, C/Domingo Miral, s/n, 50009 Zaragoza, Spain; aicisner@unizar.es (A.I.C.-G.); annikafieguthbatista@gmail.com (A.F.-B.); mirgraciamartinez@gmail.com (M.G.-M.); jaobon@unizar.es (J.O.-N.); jwhyte@unizar.es (J.W.-O.); 2 Medical and Genetic Research Group (GIIS099), Aragon Health Research Institute, 50009 Zaragoza, Spain

**Keywords:** bioanthropology, Al-Andalus necropolis, height, De Mendonça, Carrea

## Abstract

This study evaluates the agreement between Carrea’s (1920) odontological method and De Mendonça’s (2000) osteometric method for estimating height in archaeological skeletal remains from an Al-Andalus necropolis (10th–12th centuries) discovered in Zaragoza. Twenty-seven individuals (16 men, 11 women) were selected who had the femur, medial and lateral incisors, as well as the mandibular canine, fully preserved. The results showed that at the population level, both methods provide similar mean estimates of height (mean Bland–Altman differences close to zero). However, at the individual level, agreement was poor: the 95% limits of agreement reached approximately ±25 cm, and both the Lin concordance correlation coefficient (CCC = 0.140–0.156) and the Pearson cross-correlations (r = 0.156–0.171, non-significant) confirmed a weak linear relationship between the two methods. Furthermore, it was found that dental wear correlates negatively and significantly with Carrea’s estimates (r = −0.428; *p* = 0.026), suggesting that it constitutes a source of bias specific to this method. Greater dispersion of Carrea’s estimates was also observed in males, possibly linked to their higher degree of dental wear. These results indicate that although Carrea’s method may be useful for estimating the average height of a population, its applicability as an individual estimator is limited.

## 1. Introduction

The reconstruction of biological profiles—age, sex, height and ancestry—is one of the main tools for the analysis of human remains, both in the fields of physical and forensic anthropology [1,2]. Among these parameters, height plays a central role: as well as providing identifying information, it allows us to infer aspects relating to the living conditions, nutrition, and health status of past populations.

Traditionally, height is estimated through anatomical reconstruction and osteometric measurements of the long bones, with the femur being one of the most reliable indicators due to its high correlation with body height [3]. However, this approach is limited in contexts where skeletal remains are fragmented, incomplete or poorly preserved—a common occurrence at archaeological sites. Given these limitations, the study of dental structures has become increasingly important owing to the high resistance of enamel and dentine to taphonomic processes, which makes teeth a particularly useful resource for estimating body height when the postcranial skeleton is incomplete or absent [3,4].

It is within this framework that the odontological method proposed by Ubaldo Carrea in 1920 is situated; this method estimates height based on measurements of the lower incisors and canine, yielding a height range (minimum–maximum) that is particularly useful when long bones are unavailable [5].

However, the original method has limitations associated with population variability and the simplification of its mathematical models; consequently, various studies have proposed modifications that improve its accuracy by incorporating multiple odontometric variables [6,7,8]. However, most of these studies have evaluated it against actual or self-reported height, with little evidence regarding its agreement with reference osteometric methods based on long bones.

The sample analysed in the present study comes from an Al-Andalus necropolis discovered in 2023 and excavated over two archaeological campaigns in subsequent years, from which 236 individuals have been recovered to date. The burials, arranged in the right lateral decubitus position and oriented towards Mecca, conform to Islamic funerary tradition (darih or lahd type) and date from the 10th to the 12th centuries. Bioarchaeological analysis is currently underway.

This context offers a particularly suitable opportunity to assess the usefulness of Carrea’s method, given that in many of the recovered individuals, the state of conservation of the postcranial skeleton varies, whilst the teeth tend to be better preserved.

The aim of the present study is to assess the degree of agreement between height estimates obtained using Carrea’s odontological method (1920) and those obtained using Mendonça’s osteometric method (2000) applied to the femur, in a sample of 27 individuals from the Saraqusta necropolis preserving both a complete femur and intact mandibular teeth. In this way, the study aims to contribute to an assessment of the practical utility of Carrea’s method as an alternative for estimating height in archaeological contexts where long bones are absent or fragmented, but teeth are well preserved.

## 2. Materials and Methods

### 2.1. Sample

The study sample consisted of 27 adult individuals from the José Luis Pomarón Street necropolis (Zaragoza), dating from the Al-Andalus period (10th–12th centuries), selected from a total of 236 individuals.

Biological profile estimation was conducted at the Department of Human Anatomy and Histology (University of Zaragoza) by anatomists and anthropologists with extensive experience in this type of study. All observations were carried out by three observers (one anthropologist, one senior anatomist, and one junior anthropologist).

Sex estimation was based on the morphological assessment of diagnostic sexually dimorphic traits (supraorbital ridge, mastoid process, nuchal crest development, supraorbital margin, and mental eminence) following the standard criteria of Buikstra & Ubelaker, 1994 [9].

Age-at-death was estimated through the evaluation of ectocranial suture closure [9,10] and palatal suture (incisive, intermaxillary, interpalatine, and palatomaxillary) obliteration following the Mann criteria [11] because only the skull was available.

All individuals included retained the three teeth required for the application of Carrea’s method (mandibular central incisor, lateral incisor, and canine) in an intact and measurable condition without diastemata, crowding, or malposition; no measurements were taken on partial or incomplete teeth.

### 2.2. Estimation of Reference Height

The reference height for each individual was calculated using the linear regression formulas proposed by Mendonça [12] for a 20th-century Portuguese population; these may therefore be applicable to the population in our study by geographical extension rather than by time period.

The femur was chosen as the reference bone over other long bones, given that height estimation methods based on the femur provide results closest to actual height [3], with confidence intervals significantly narrower than those for other bone structures (±5.96 cm in women and ±6.96 cm in men). The maximum length of the femur was measured for this purpose. The height obtained using these formulae corresponds to the standard estimate, valid in 95 per cent of cases, and was taken in this study as the reference method (*gold standard*) against which Carrea’s method was evaluated.

Given that the Carrea method does not yield a single height value but rather a range (minimum height–maximum height), and in order to ensure that both methods were comparable, Mendonça’s height was not used as a single value but was likewise expressed as a range by subtracting and adding the margin of error inherent in the original formula (±5.96 cm for women and ±6.96 cm for men, depending on the individual’s sex) to the specific height: specific height − margin of error, and specific height + margin of error. Thus, Mendonça_min and Mendonça_max represent the lower and upper limits of this interval, and are the values actually used in the comparisons and in the analysis of concordance with Carrea’s method.

### 2.3. Dental Measurement

Dental measurements were carried out on the 27 selected individuals using a digital calliper, in accordance with Carrea’s method, on the mandibular central incisor, lateral incisor and canine, depending on the availability of each tooth in each individual.

The two measurements used in the method are the arch and radius-chord. The arch corresponds to the sum of the mesiodistal diameters of the lower central incisor, lateral incisor, and canine on the same hemiarch, measured from their vestibular surface, in millimetres [5,13]. The chord radius corresponds to the chord of that arch and was derived using a conversion coefficient (chord radius = arch × 0.94), in accordance with the geometric relationship between the dental arch and its chord described by Carrea [5,13]. According to the original formulation, the result obtained from the arch approximates the expected estimate for males, whilst the result obtained from the radius–chord ratio approximates that expected for females [13,14]; however, in the present study, both values were calculated for the entire sample, regardless of sex, in order to obtain, in all cases, a range (minimum–maximum) comparable to the range derived from Mendonça’s method.

Maximum height (cm) = [(arch (mm) × 6 × 3.1416 × 10)/2]/10

Minimum height (cm) = [(radius-chord (mm) × 6 × 3.1416 × 10)/2]/10

Thus, the Carrea method does not yield a single point estimate of height, but rather a range (minimum–maximum), which influenced the design of the statistical analysis used to compare it with Mendonça’s point estimate.

Dental wear, as a discriminating factor, was recorded using a combined system based on the Brothwell [15] and Zoubov [3,16] scales. A scale ranging from 0 (no wear) to 6 (excessive wear) was established according to the Brothwell method for the study of molars, and from 0 (no wear) to 5 (excessive wear) according to the Zoubov method for the remaining teeth. The mean value obtained from both scales was used to estimate dental wear in each individual.

### 2.4. Statistical Analysis

The data were analysed descriptively to characterise the general behaviour of the sample. For continuous quantitative variables, measures of central tendency (arithmetic mean) and dispersion (standard deviation, minimum and maximum values) were calculated. The normality of the distribution is assessed using the Shapiro–Wilk test.

To assess whether the Carrea method constitutes a valid alternative to the Mendonça method, an inter-method agreement analysis was carried out rather than a simple comparison of means, given that two methods may yield similar average values and yet differ significantly at the individual level. The analysis was organised in three stages:

1. Overall agreement between methods

The difference between methods (Mendonça − Carrea) was calculated for the minimum and maximum limits of the Carrea interval. Bland–Altman analysis was applied to obtain the mean bias (with its 95% confidence interval) and the 95% limits of agreement (mean bias ± 1.96 × SD).

2. Agreement by sex

The magnitude of the discrepancy (Mendonça–Carrea) between men and women was compared using the Student’s *t*-test for independent samples (or the Mann–Whitney U test, depending on whether the normality criterion was met). The Bland–Altman plot analysis was repeated, stratified by sex.

3. Relationship with dental wear

We analysed whether the degree of dental wear, commonly used as an indicator of biological age, was associated with the magnitude or direction of inter-method error. To this end, Pearson’s and/or Spearman’s correlation coefficients (depending on normality) were calculated between dental wear and the difference (Mendonça − Carrea), considering both the signed value and the absolute value.

All statistical analyses were carried out using Jamovi^®^ software 2.6.26 version. For all inferential tests, the level of statistical significance was set at *p* < 0.05.

## 3. Results

### 3.1. Sample Description: General Composition

Of the total 27 individuals, all of whom were adults, 59% (16/27) was classified as male and 41% (11/27) as female. The overall preservation index of the sample, following the criteria established by Bello [17], was classified as class 3 (25–49% of the skeleton preserved).

Dental wear, recorded using a combined system of the Brothwell [15] and the Zoubov scale [3,16], and assessed independently of the height calculation, showed differences between the sexes: the male group had a mean of 3.22 (SD = 1.40; range 0–5), which was higher than that observed in the female group, with a mean of 2.64 (SD = 1.87; range 0–6).

### 3.2. Height Estimates by Method

**Figure 1:**
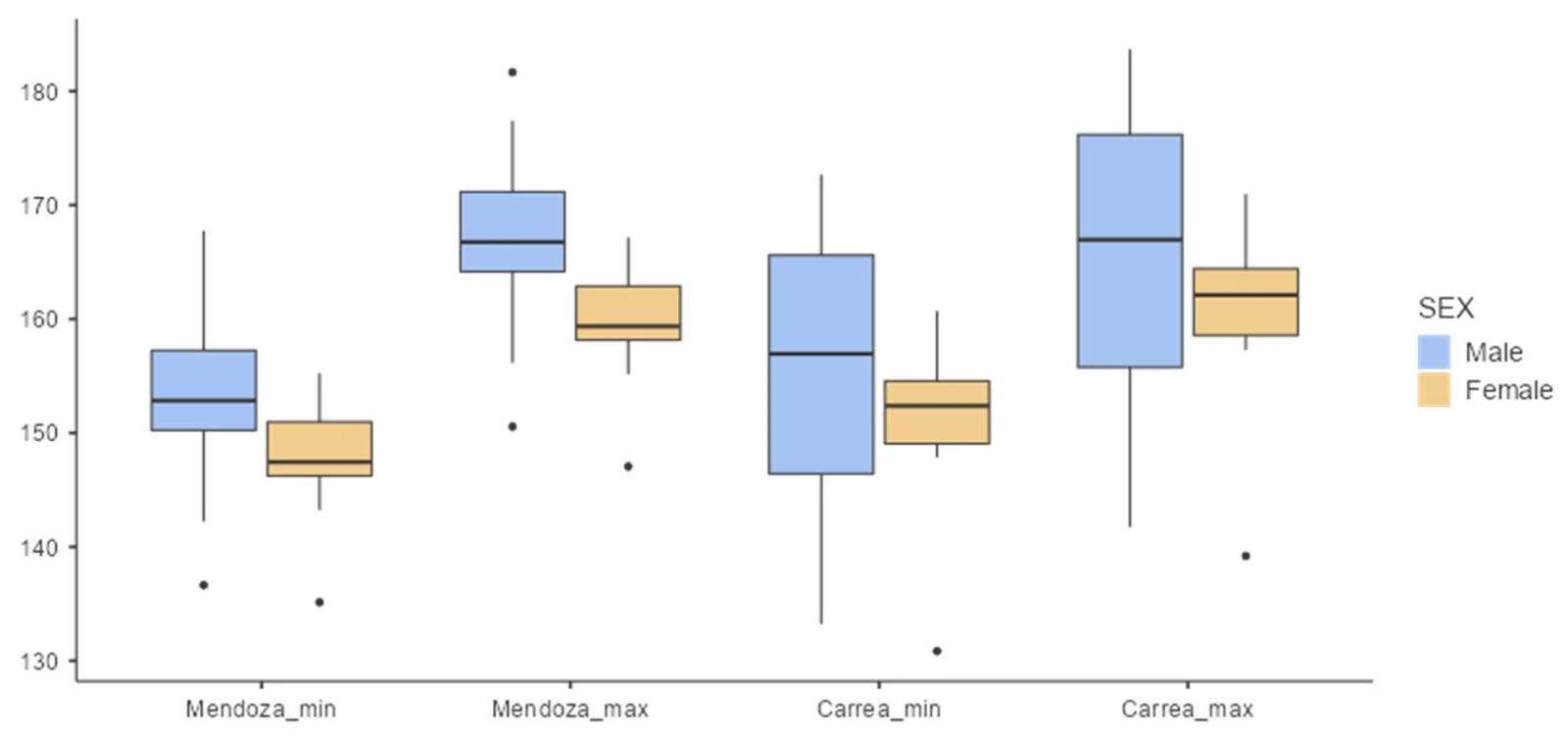
Height estimates by method and sex.

The estimated height values using the Mendonça method ranged from a mean of 151 cm (minimum) to 164 cm (maximum), with standard deviations of 7.33 cm and 7.76 cm, respectively. Using the Carrea method, the means ranged from 153 cm (minimum) to 163 cm (maximum), with a slightly greater dispersion (SD = 11.0–11.7 cm) (Figure 1). When broken down by sex, the male group showed consistently higher estimates than the female group in both methods, and the range of Carrea’s estimates (SD = 12.8–13.7 in men; 7.66–8.15 in women) was considerably greater than that of the Mendonça method (SD = 7.70 and 5.69, respectively) in both groups (Figure 1).

### 3.3. Analysis of the Sample

#### 3.3.1. Normality of the Variables

The Shapiro–Wilk test revealed no significant deviations from normality in any of the four height variables analysed (*p* > 0.05 in all cases). For the variables derived from the Mendonça method, the W values ranged from 0.983 to 0.993 (*p* = 0.923–0.999), whilst for the two variables from the Carrea method, a W value of 0.957 was obtained in both cases (*p* = 0.308). As no deviations from normality were detected, parametric statistics were used in the subsequent inferential analyses at a general level.

#### 3.3.2. Analysis of Agreement Between Methods

Given that the analysis of means alone does not allow us to determine whether two methods are interchangeable at the individual level, a Bland–Altman plot was applied, with the difference defined as (Mendonça − Carrea).

**Figure 2:**
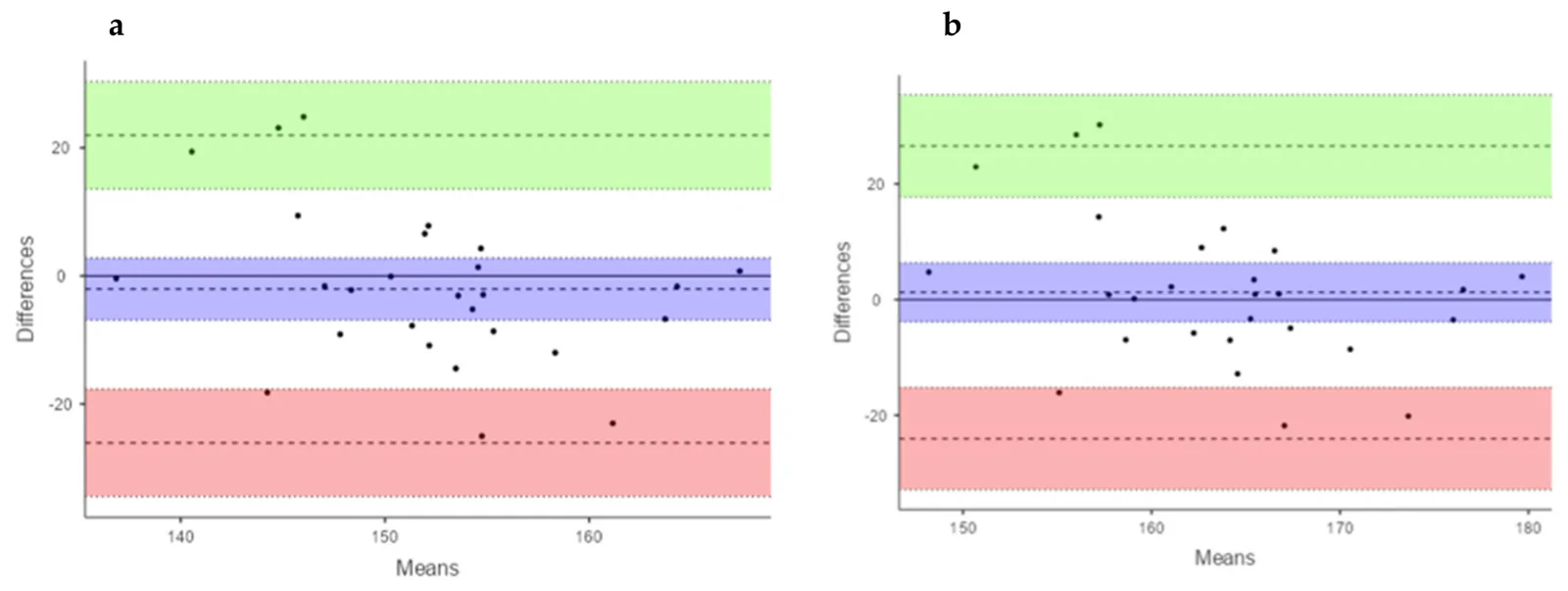
Bland–Altman plot for agreement between de Mendonça and Carrea, calculated for the lower limit (**a**) and the upper limit (**b**). Mean and estimated differences in cm.

Figure 2 shows the Bland–Altman plot for the agreement between the Mendonça method and the Carrea method, calculated separately for the lower limit (a) and the upper limit (b) of the Carrea interval. For the lower limit (Figure 2a), the mean difference (Mendonça − Carrea) was −2.06 cm (95% CI: −6.91 to 2.79), with 95% agreement limits ranging from −26.08 cm (95% CI: −34.47 to −17.69) to 21.96 cm (95% CI: 13.57 to 30.35). For the upper limit (Figure 2b), the bias was 1.26 cm (95% CI: −3.85 to 6.37), with 95% agreement limits ranging from −24.04 cm (95% CI: −32.88 to −15.21) to 26.57 cm (95% CI: 17.73 to 35.41). The bias was close to zero, indicating that, although on average the two methods do not differ systematically, individual differences can reach up to approximately ±25 cm, suggesting poor case-by-case agreement.

**Figure 3:**
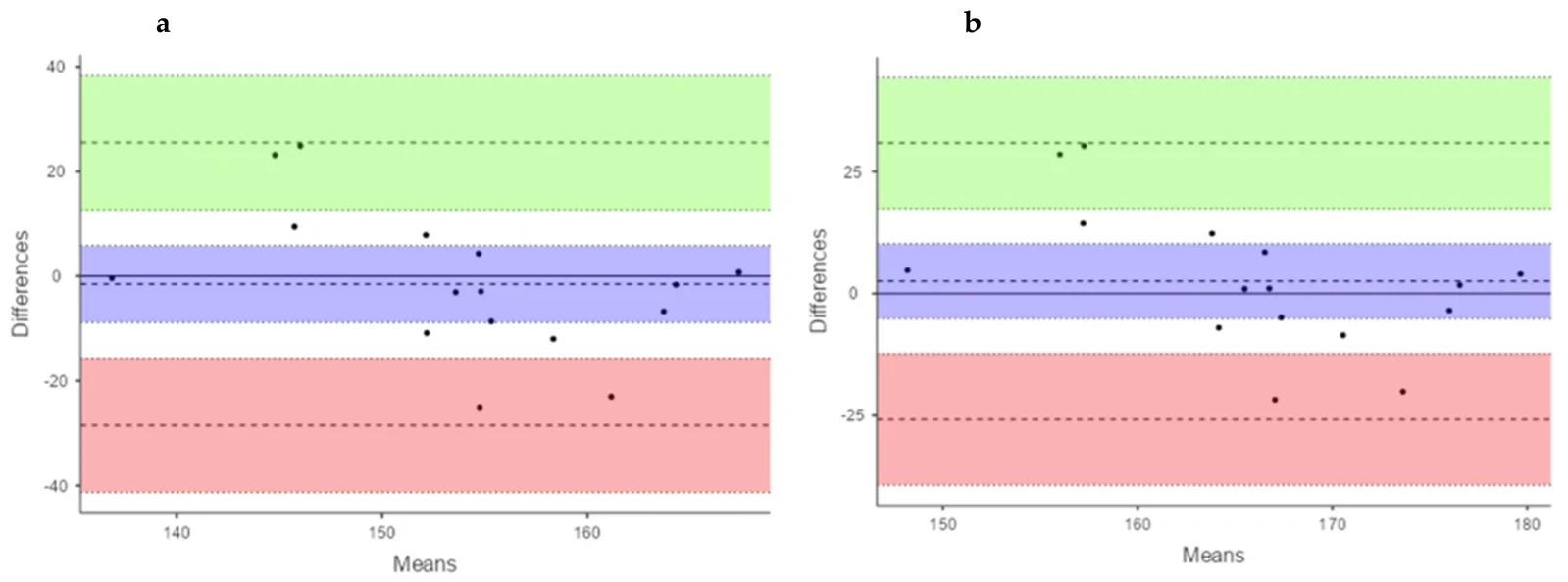
Bland–Altman plot for agreement between de Mendonça and Carrea, calculated for the lower limit (**a**) and the upper limit (**b**) in male subjects. Mean and estimated differences in cm.

**Figure 4:**
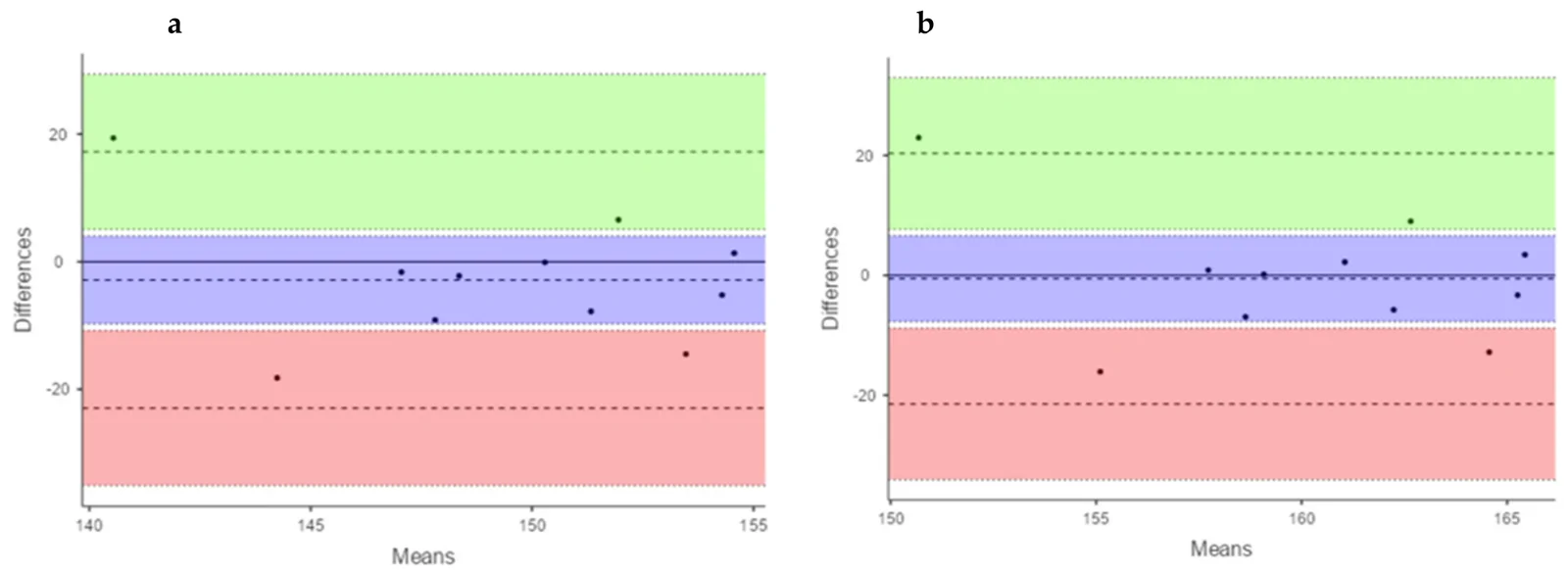
Bland–Altman plot for agreement between the de Mendonça and Carrea methods, calculated for the minimum limit (**a**) and the maximum limit (**b**) in female subjects. Mean and estimated differences in cm.

When stratified by sex, the pattern remained the same. In the male group (*n* = 16), the bias was −1.51 cm (95% CI: −8.85 to 5.82) for the minimum (Figure 3a) and 2.52 cm (95% CI: −5.18 to 10.2) for the maximum, with agreement limits of −28.50 to 25.47 cm and −25.81 to 30.85 cm, respectively (Figure 3b). In the female group (*n* = 11), the bias was −2.86 cm (95% CI: −9.75 to 4.03) for the minimum and −0.57 cm (95% CI: −7.73 to 6.60) for the maximum (Figure 4a), with agreement limits of −22.95 to 17.24 cm and −21.47 to 20.34 cm (Figure 4b). In none of the four subgroups did the confidence interval for bias exclude zero; therefore, no systematic bias dependent on sex was detected. However, the limits of agreement remained wide in all cases, reflecting the lack of individual agreement estimated by sex.

To assess the agreement between the methods (Carrea and de Mendonça) in estimating the minimum and maximum values, Lin’s coefficient of concordance correlation (CCC) was calculated for the sample. The results showed a poor level of agreement between the two methods for both the minimum value (CCC = 0.140; 95% CI: −0.208 to 0.457) and the maximum value (CCC = 0.156; 95% CI: −0.197 to 0.473). In both cases, the 95% confidence intervals included the value zero, indicating that the observed agreement was not statistically significant. These findings suggest a considerable discrepancy between the two methods for estimating minimum and maximum height at the individual level.

Table 1 shows the Pearson correlation matrix between the height variables obtained by both the Mendonça and Carrea methods (minimum limit, maximum limit, and overall value). This revealed high internal consistency in both methods between their respective range limits and point values (Mendonça: r = 0.993–0.998; Carrea: r = 1.000; all with *p* < 0.001), confirming the validity of the calculation procedure for each estimation range. However, when comparing the two methods with each other, all cross-correlations were weak and not statistically significant (r = 0.156–0.171; *p* > 0.39 in all cases), regardless of whether the minimum, maximum, or specific values of each estimate were used. These results reaffirm that the height estimates based on the femur (Mendonça) and teeth (Carrea) did not show a significant linear relationship with one another in the sample analysed.

#### 3.3.3. Analysis of Agreement Between Methods According to Dental Wear

Table 2 shows the Pearson correlation matrix between the degree of dental wear and the height estimates obtained by both methods. Dental wear showed a negative, weak-to-moderate and statistically significant correlation with the minimum and maximum limits of the Carrea interval (r = −0.428; *p* = 0.026 in both cases), indicating that as the degree of dental wear increases, the height estimate provided by the Carrea method tends to decrease. These findings suggest that dental wear constitutes a source of systematic bias specific to the Carrea method, which could explain, at least in part, the weak agreement previously observed between the two estimation methods.

## 4. Discussion

### 4.1. Agreement Between the Carrea and Mendonça Methods

Estimating height from long bones, using the regression formula established by Mendonça [12], is one of the standard methods for determining a subject’s height when long bones are present. However, in certain archaeological contexts, it is not possible to recover these bones in their entirety; consequently, alternative methods such as Carrea’s have been proposed for estimating height from the mesio-distal diameter of the teeth (medial and lateral incisors and canines). In our study, we were able to confirm that the height estimation using the Carrea method [5], despite presenting a greater deviation in the estimate (SD 11.0–11.7 vs. 7.33–7.76) did not present a significant variation compared to the height estimate established from the Mendonça method when the study is carried out at the population level with the aim of estimating the average height of a population. These findings are consistent with those reported by other authors [4,18,19] when estimating height in populations using the Carrea method. This result is therefore significant because it indicates that when the aim of the analysis is to characterise the average height of a population, Carrea’s odontometric method could constitute a methodologically valid alternative in contexts where the femur is not available. However, this absence of mean bias contrasts sharply with what is observed at the individual level, where the applicability of Carrea’s method shows poor agreement when compared with the femoral standard. These findings corroborate the observations of other authors who have noted that individual height estimates vary considerably depending on the dental arch, the type of dentition, and the reference population [4,18,20,21].

### 4.2. Influence of Dental Wear and Sex on the Estimate

A significant contribution of our study is the identification that dental wear—assessed using the combined scale of Brothwell (1989) and Zoubov (1968), as cited by Krenzer (2006) [3,15,16]—correlates negatively and significantly with Carrea’s estimates, and given that both wear scales used are well-established in bioarchaeology for estimating the age of adult individuals in European archaeological contexts, this should be considered to avoid underestimating height as a result of wear.

When the results were broken down by sex, it was observed that the male group exhibited a systematically higher degree of dental wear than the female group (mean = 3.22 vs. 2.64), although with greater variation among female subjects (SD = 1.87 vs. 1.40). This difference is consistent with findings from other archaeological populations where the degree and pattern of dental wear tend to vary between the sexes, reflecting a sexual division of labour relating to diet, food preparation, or the extramasticatory use of the dentition [22,23,24,25].

Secondly, both methods of estimating height yielded systematically higher values in the male group than in the female group, which is to be expected given the widespread sexual dimorphism in body and bone size, indicating that this results in larger teeth in men than in women, as described by authors such as Acharya and Mainali, Garn et al., and Pettenati-Soubayroux et al. [26,27,28].

However, it is particularly striking that the range of Carrea’s estimates was notably greater in the male group (SD = 12.8–13.7 cm) than in the female group (SD = 7.66–8.15 cm), whereas in Mendonça’s method, this difference between the sexes was much less pronounced (SD = 7.70 cm in men vs. 5.69 cm in women). This greater variability observed by Carrea in men could be related, at least in part, to the greater degree of dental wear observed in this group, insofar as more intense and heterogeneous wear introduces an additional source of error into the mesiodistal measurements used by the method, as highlighted by studies of archaeological populations documenting sexual variability in the degree and pattern of dental wear depending on diet and subsistence mode.

### 4.3. Population and Methodological Variability of the Carrea Method

The available studies show that the validity of the Carrea method is far from universal, and that its performance depends to a large extent on the population and the type of dentition studied. Whilst the method has yielded favourable results in mestizo samples from Ecuador [14] and Peru [8], the Ecuadorian study itself notes that its reliability tends to decrease in non-mestizo populations, such as indigenous groups [14]. Similarly, in an Indian population, Rekhi et al. [21] documented that the type of dental alignment strongly influences the results: the method works reasonably well in normal or crowded dentitions, but loses accuracy markedly in the presence of diastemas, as these alter the chord value, and consequently, the estimated height range. This same limitation was documented in a Brazilian population by Lima et al. [19,29], who also noted that the method, in its original formulation, is practically inapplicable to the maxillary arch (with error rates approaching 100 per cent), leading to the subsequent development of alternative mathematical denominators for that application [6].

Another important aspect highlighted in the literature is the impact of the measurement technique on the accuracy of the method. Cavalcanti et al. [20] showed that the original method, using a millimetre tape measure, achieved an accuracy of only between 36% and 48% in their sample, whilst a modified version using more precise instruments (a dry-point compass and a ruler) increased the accuracy to 96%. This suggests that much of the variability reported in the literature—and possibly also part of the lack of agreement observed in our study—could be associated not only with population differences, but also with the instrument and measurement protocol used, an aspect that was not varied in the present study but which should be considered in future studies using this same Al-Andalus sample.

Finally, it is worth noting that our study may be the first to evaluate Carrea’s method against a reference osteometric method [12] in an archaeological population dating from the medieval Al-Andalus period, a context that differs substantially from those addressed in the previous literature—which has focused mainly on living populations or recent cadavers from contemporary forensic contexts. The lack of equivalent studies on archaeological populations currently prevents us from determining whether the weak concordance observed here is due to characteristics specific to this particular population or whether it extends to other bioarchaeological contexts.

### 4.4. Limitations

It should be noted that the sample size used (*n* = 27, with an uneven distribution between the sexes: 16 men and 11 women) limits the statistical power of the study, particularly when it comes to detecting smaller differences between subgroups; therefore, the results regarding concordance and comparisons by sex should be interpreted with caution and confirmed in larger samples. Furthermore, as there was no independently verified measure of actual height in the sample, it was not possible to determine which of the two methods came closest to the true value; rather, only their relative agreement with one another could be assessed. Finally, the observed relationship between dental wear and the direction of bias between methods is correlational in nature; therefore, it does not allow for the establishment of causality, and future studies should incorporate a regression analysis adjusted for confounding variables (such as chronological age) to characterise this finding more precisely.

## 5. Conclusions

Carrea’s method could prove to be a reasonable tool for estimating the average height of a population when the femur is not available; however, it is not reliable as an individual estimator, given its poor agreement with the reference method established by Mendonça based on the femur. Furthermore, dental wear appears to systematically underestimate estimated height (the greater the dental wear, the shorter the estimated height). These findings, obtained in a medieval Al-Andalus archaeological context, should be interpreted with caution due to the limited sample size and the absence of verified actual height; therefore, future studies with larger samples are required to confirm their validity in similar bioarchaeological contexts.

## Figures and Tables

**Table 1:** Correlation matrix between the height values obtained by both the Mendonça and Carrea methods (minimum limit, maximum limit, and overall value).

Matrix Correlation						
		Mendonça_Min	Mendonça_Max	Carrea_Min	Carrea_Max	Mendonça
Mendonça_min	Pearson’s R	—				
	Gl	—				
	*p* value	—				
Mendonça_max	Pearson’s R	0.993	—			
	Gl	25	—			
	*p* value	<0.001	—			
Carrea_min	Pearson’s R	0.156	0.171	—		
	Gl	25	25	—		
	*p* value	0.437	0.393	—		
Carrea_max	Pearson’s R	0.156	0.171	1.000	—	
	Gl	25	25	25	—	
	*p* value	0.437	0.394	<0.001	—	
Mendonça	Pearson’s R	0.998	0.998	0.164	0.164	—
	Gl	25	25	25	25	—
	*p* value	<0.001	<0.001	0.413	0.413	—

**Table 2:** Correlation between dental wear and estimated height.

Correlation Matrix				
		Carrea_Min	Carrea_Max	Dental Wear
Carrea_min	Pearson’s R	—		
	Gl	—		
	*p* value	—		
Carrea_max	Pearson’s R	1.000	—	
	Gl	25	—	
	*p* value	<0.001	—	
Dental wear	Pearson’s R	−0.428	−0.428	—
	Gl	25	25	—
	*p* value	0.026	0.026	—

## Data Availability

The original contributions presented in this study are included in the article. Further inquiries can be directed to the corresponding author.

## References

[1] Adams B.J. (2009). Forensic Anthropology.

[2] Burns K.R. (2012). Forensic Anthropology Training Manual.

[3] Krenzer U. (2006). Compendium of Forensic Anthropological Methods for the Reconstruction of the Osteobiological Profile.

[4] Garrido Y., Zavando D., Suazo Galdames I. (2012). Estimation of height based on the dimensions of the deciduous dentition. Int. J. Odontostomatol..

[5] Carrea J.U. (1920). Ensayos Odontométricos.

[6] Lima L.N.C., Tinoco R.L.R., Picapedra A., Sassi C., Ulbricht V., Schmidt C.M., Daruge Júnior E. (2017). Estimation of stature using the upper dental arch: A modified version of Carrea’s method. Int. J. Odontostomatol..

[7] Rangari P., Singh U., Singh N., Kamdi S.G. (2018). Stature estimation and its reliability in different types of dental alignment using the Carrea index. Int. J. Med. Res. Rev..

[8] De Los Rios E., Barriga M. (2015). Estimation of actual and estimated height using the mesio-distal diameters of the central and lateral incisors and lower canines, according to Carrea’s method, in young people from Arequipa. Int. J. Med. Surg. Sci..

[9] Buikstra J.E., Ubelaker D.H. (1994). Measurement of adult remains. Standards for Data Collection from Human Skeletal Remains: Proceedings of a Seminar at the Field Museum of Natural History.

[10] Meindl R.S., Lovejoy C.O. (1985). Ectocranial suture closure: A revised method for the determination of skeletal age at death based on the lateral-anterior sutures. Am. J. Phys. Anthropol..

[11] Mann R.W., Symes S.A., Bass W.M. (1987). Maxillary suture obliteration: Aging the human skeleton based on intact or fragmentary maxilla. J. Forensic Sci..

[12] De Mendonça M.C. (2000). Estimation of height from the length of long bones in a Portuguese adult population. Am. J. Phys. Anthropol..

[13] Briñón J.M. (1984). Odontología Legal y Práctica Forense.

[14] Arízaga J.E.B., Manzano V.P.C., Pazmiño M.D.L.A.G., Yánez J.M.Q., Machado J.P. (2022). Application of Carrea’s odontological method for forensic identification. Relationship between actual and estimated height. Anatomía Digit..

[15] Brothwell D.R. (1989). The relationship of tooth wear to aging. Age Markers in the Human Skeleton.

[16] Zoubov A.A. (1968). Dentistry. Methodology of Anthropological Research.

[17] Bello S.M., Thomann A., Signoli M., Dutour O. (2006). The reproducibility of skeletal preservation state metrics in bioarchaeology. Int. J. Osteoarchaeol..

[18] Gajardo P., Gajardo M., Torres S., Zavando D., Suazo Galdames I. (2011). Determination of height based on the maxillary arch and maxillary radius-chord. Int. J. Odontostomatol..

[19] Lima L.N.C., Neves G.L.S., Rabello P.M. (2008). Carrea’s index in dental students at the Federal University of Paraíba. Braz. J. Oral Sci..

[20] Cavalcanti A.L., Porto D.E., Maia A.M.A., Melo T.R.N.B. (2007). Stature estimation by using the dental analysis: Comparative study between Carrea’s and the modified methods. Rev. Odontol. UNESP.

[21] Rekhi A., Marya C.M., Nagpal R., Oberoi S.S. (2014). Estimation of stature in a young adult Indian population using the Carrea’s index. J. Forensic Odonto-Stomatol..

[22] Acharya A.B., Prabhu S., Muddapur M.V. (2011). Odontometric sex assessment from logistic regression analysis. Int. J. Leg. Med..

[23] Luna L.H. (2008). Demographic structure and patterns of dental wear in the bone sample from the Chenque I site (Lihué Calel National Park, Argentina). Rev. Argent. Antropol. Biol..

[24] Teschler-Nicola M., Prossinger H., Alt K.W., Rösing F.W., Teschler-Nicola M. (1998). Sex determination using tooth dimensions. Dental Anthropology.

[25] Viciano J., De Luca S., Alemán I. (2013). Odontometric sex estimation in a contemporary Spanish population. Int. J. Leg. Med..

[26] Acharya A.B., Mainali S. (2007). Univariate sex dimorphism in the Nepalese dentition and the use of discriminant functions in gender assessment. Forensic Sci. Int..

[27] Garn S.M., Lewis A.B., Kerewsky R.S. (1967). The relationship between sexual dimorphism in tooth size and body size as studied within families. Arch. Oral Biol..

[28] Pettenati-Soubayroux I., Signoli M., Dutour O. (2002). Sexual dimorphism in teeth: Discriminatory effectiveness of permanent lower canine size observed in an 18th-century osteological series. Forensic Sci. Int..

[29] Lima L., da Costa Y., Tinoco R., Rabello P., Daruge Júnior E. (2011). Stature estimation using Carrea’s index and its reliability in different types of dental alignment. J. Forensic Odonto-Stomatol..

